# Completed Suicide with Violent and Non-Violent Methods in Rural Shandong, China: A Psychological Autopsy Study

**DOI:** 10.1371/journal.pone.0104333

**Published:** 2014-08-11

**Authors:** Shi-Hua Sun, Cun-Xian Jia

**Affiliations:** 1 Department of Epidemiology and Health Statistics, Shandong University School of Public Health, Jinan, China; 2 Shandong University Center for Suicide Prevention Research, Jinan, China; University of Vienna, Austria

## Abstract

**Background:**

This study aims to describe the specific characteristics of completed suicides by violent methods and non-violent methods in rural Chinese population, and to explore the related factors for corresponding methods.

**Methods:**

Data of this study came from investigation of 199 completed suicide cases and their paired controls of rural areas in three different counties in Shandong, China, by interviewing one informant of each subject using the method of Psychological Autopsy (PA).

**Results:**

There were 78 (39.2%) suicides with violent methods and 121 (60.8%) suicides with non-violent methods. Ingesting pesticides, as a non-violent method, appeared to be the most common suicide method (103, 51.8%). Hanging (73 cases, 36.7%) and drowning (5 cases, 2.5%) were the only violent methods observed. Storage of pesticides at home and higher suicide intent score were significantly associated with choice of violent methods while committing suicide. Risk factors related to suicide death included negative life events and hopelessness.

**Conclusions:**

Suicide with violent methods has different factors from suicide with non-violent methods. Suicide methods should be considered in suicide prevention and intervention strategies.

## Introduction

Suicide has become a severe public health problem in the world [1]. In China, suicide ranks as fifth among the leading causes of death in the whole population and ranks the first in the 15–34 age-groups [2]–[4]. According to the 2011 China Health Statistical Yearbook [5], suicide rate in rural China by 2010 was 10.01/100,000, and suicide rate in urban China was 6.86/100, 000. With the rural population accounted for 50.1% of the whole population in 2010, the issue of suicide has been more serious in rural China.

Researchers have identified the remarkable differences of certain patterns of suicide methods observed in different countries. Some studies [6]–[12] have found that choice of certain methods while committing suicide was not made randomly, but was influenced by some fixed factors. Availability and social acceptability of certain methods were commonly included in such factors. Also, different areas around the world witnessed different leading methods that were highly limited to local communities.

Some possible determinants for different methods of suicide such as age, gender, suicide intent, mental health status, timing, and so forth have been identified by researchers. One study [8] conducted in New York has found that although the most common method of suicide in America was firearm shots, fall from height was a highly used method by elderly residents. A study [10] for a Japanese population found that men were more likely to use lethal methods while committing suicides than women, which is similar to findings of studies on European populations [9], [13]. Furthermore, it was found that the method used at an unsuccessful suicide attempt may predict later completed suicide [14], suggesting study on suicide methods is of much significance in suicide prevention. Suicide methods are mostly divided into two main categories [12], [15], [16]: firearm or shotgun, hanging, cutting and piercing with sharp objects, jumping from high places, and getting run over by train or other vehicles are classified as violent methods; while ingestion of pesticides, poison by gases, suffocation and overdose are classified as nonviolent methods.

As a non-violent suicide method, ingesting pesticide has been reported as the leading method of suicide in rural China [2], [4], [17], [18]. On the other hand, violent methods have particularly been highlighted because of higher lethality and stronger evidence of the cases' mental disorder and self-harm determination [6], [9], [11], [12].

Study on suicide methods proved to be of special significance in suicide prevention among various populations [14], [19]–[21]. And it was found that the method used at an unsuccessful suicide attempt may predict later completed suicide [14]. Yet counterpart researches were extremely limited on Chinese suicide cases. Conner and Phillips' team [22] conducted a study on suicides in a sample recruited from multiple cites of China, and revealed that low-planned (impulsive) suicides, which were believed to be quite common among Chinese suicides, were associated with the method of ingesting pesticides stored at home. But their study did not analyze comprehensive information about violent vs. non-violent suicide methods and possible correlates profiling suicide cases socially or psychologically. Furthermore, the limited sample size expected to represent both rural and urban China might fail to exactly picture the situation of suicide in rural Shandong Province, considering the even less cases recruited from each research site. In this study, we aimed to describe the specific characteristics of suicide with violent methods vs. non-violent methods in rural Shandong Province, China and to explore possible risk factors related to suicides with violent methods and without.

## Methods

### Study Population

This study was designed as a paired case-control study [23]. We recruited suicide cases that happened between September 1^st^, 2008 and August 31^st^, 2009 from 25 towns in rural Shandong Province, China. All the 25 towns were chosen from three counties which are disease surveillance points of Shandong Province: Zoucheng, Ju'nan, and Gaotang. We recruited 219 cases at the beginning yet 19 of them refused to participate in this study. Ultimately 200 suicide cases and their paired controls were obtained for analysis by trained interviewers. Of all the 200 cases interviewed, 199 reported identified suicide methods. Each suicide case was paired with a control with the same gender, same age (±3 years), same residence, and without previous suicide attempts. Information of subjects was collected by interviewing one qualified informant of each case and each control, using the method of Psychological Autopsy (PA), which has long been attested to be valid and feasible in terms of typical Chinese cultural and social circumstances [3], [4], [17], [24], [25]. As the only possible way to reconstruct the mental and psychological status of subjects before committing suicides, PA was proved to be valid in our data, too, of which further detail could be referred to the work by Lu et al.[23].

### Procedures

The informants of both the cases and paired controls were chosen with the help of village doctors or local government officials who were familiar with the local residents and environments. Priority of selecting informants for each subject was to their family relatives, then to their close friends or neighbors. Interview was conducted between 2–6 months after each completed suicide event to obtain more objective and valid information about the subjects. The protocol of this study was reviewed and approved by the ethics committee of Shandong University School of Public Health. Written informed consents were obtained before each interview. Quality control measurements were taken to assure the interview environments to be without interruption and the interviewing time to be averaged 1.5 h.

### Instruments

#### (1) Social-economic information

Between 2–6 months after the suicide events, the informant of each subject was contacted to participate the psychological autopsy interview conducted by trained interviewers. The complete questionnaire included data of social-economic factors, health status, psychological factors, and suicide behavior related characteristics.

Social-economic factors included age, gender (female = 0; male = 1), marriage status (never married: single and no dating; ever married: married, widowed, divorced, remarried and single but dating with others), education years, personal annual income (RMB), religion (no: atheist; yes: Taoism, Islam, Protestantism, Catholicism, Buddhism, other), family history of suicide, previous attempts of suicide, and pesticides storage at home.

#### (2) Medical history - physical and mental problems

Health status factors included both physical illnesses and mental illnesses. Physical illness status was obtained based on the question “Do you know whether he/she has ever had serious or chronic illness?” The Chinese version of the Structured Clinical Interview for the DSM-IV Axis I Disorders (2002 Edition) [3], [4], [25] was used to diagnose mental disorders, which has been widely used in psychological autopsy studies under various Chinese circumstances and proved to be trustworthy [3], [12], [17], [23], [26].

#### (3) Psychological status - before committing suicide

Psychological factors were obtained by various instruments.

The Chinese version of Interview of Recent Life Events (IRLE) [27]–[29] scale with 64 items, which was modified based on the original scale by Paykel according to Chinese rural conditions, to identify life events in the past one year before suicide deaths concerning the following 5 aspects: marriage events (14 items), family issues (18 items), work and study events (10 items), health related events (10 items), and law related issues (9 items). Each life event identified by the scale were evaluated with 5 dimensions including timing, frequency, feature (good or bad), extent of effect on the subjects' mental status (with 5 levels from 0 = of no influence at all to 5 = of extremely significant influence), and lasting period of mental effects. In this study, the Negative Life Events variable was categorized into 4 levels: 0 = no event, 1 = 1 event, 2 = 2 events, and 3 = 3 or more events.

The Social Support Rating Scale (SSRS) [30]–[32], which is a 10-item scale aimed to evaluate the general or specific supportive resources obtained by subjects from surrounding social environments. This scale consists of three sub-categories: objective support with 3 items (item 2, 6, and 7), subjective support with 4 items (item 1, 3, 4, and 5), and usage extent of social support with 3 items (item 8, 9, and 10). Total score of the SSRS is calculated by adding all the scores of each item, and higher scores means better social support resource.

The Chinese version of the first eight items of the Beck Suicide Intent Scale (SIS) [33]–[35], which is called as the Chinese shortened version of suicide intent scale (C-SIS), was used to evaluate suicide intent of the suicide victims. The total score can range from 0 to 16. Higher score means stronger suicide intent.

A hopelessness scale was employed in this study modified based on the original Beck Hopelessness Scale (BHS) [36]–[38] to evaluate subjects' negative ideas, negative expectations and pessimistic moods, thus indicating the extent of pessimistic expectations of subjects toward reality and the future. The BHS was adjusted with the standard of each item to report status of subjects 1 week before suicide deaths/interview time instead of 1–2 weeks and answers for each item vary from 1 (completely fit me) to 5 (completely opposite to me). The total score can range from 20 to 100.

We used the Spielberger State-Trait Anxiety Inventory (STAI) which was first introduced by Spielberger in 1983 [39], [40] to evaluate subjects' anxiety mood. The Chinese version has been widely used among Chinese populations and has been proved to be valid [12], [23], [25]. The scale has 20 items, with 9 items representing positive mood and 11 items representing negative mood. Each item has four alternative answers from never = 1 to always = 4, and the total score ranges from 20 to 80. Higher score means higher extent of anxiety mood.

The adjusted Barratt Impulsivity Scale (BIS-11) Chinese version [23], [41]–[43] was used to measure the impulsivity, a 30-item scale translated by Li and Phillips. The Chinese version consists of three sub-scales: plan scale (negative scale), action scale (positive scale), and cognition scale (negative scale), with answers for each item ranging from no = 1, seldom = 2, sometimes = 3, often = 4, to always = 5. Total scores of each sub-scale are calculated as following: original scores are obtained by adding up original scores of all items of each sub-scale, ranging from 10–40, then original score of each sub-scale is calculated with the formula *[(Σ score of items −10)/40] × 100* to get a 0–100 scale score. Final score of the impulsivity scale is obtained by *(Σ score of sub-scale)/3*. Higher score means higher degree of impulsivity.

The Chinese version of the 10-item Rosenberg (1965) Self-Esteem Scale (SES), which was first translated by Ji and Yu into Chinese and has been one of the most widely used scales to evaluate self-esteem in China [43]–[46]. Item 3, 5, 9, 10 are negative items. Each positive item is valued from 1–4 and each negative item from 4–1. The total score ranges from 10 to 40. Higher score means higher extent of self-esteem.

All the psychological instruments employed in this study have been proved to be valid and reliable in previous studies among Chinese populations, with reliability and validity of the scales discussed in other researches, some of which were also based on our database [23], [25], [26], [32], [33], [35], [37], [38], [46], [47].

#### (4) Suicide behavior characteristics

A semi-structured interview for each informant was conducted to identify information concerning characteristics of the suicide behavior, including causes, timing, location and method of suicide behavior.

### Statistical Analysis

We used the Statistical Package for Social Sciences (SPSS for Windows, version 16.0, SPSS Inc., Chicago, IL) in the statistical analyses process. Features including frequency and percentage of suicide methods observed in this study were described by category, gender, location, season, and time. Chi-square tests were used to compare categorical variables of demographic or health status between violent methods and nonviolent methods. T-tests or Kolmogorov–Smirnov Z-tests were used to compare continual variables such as personal annual income, education years, social support, and scores of psychological status between suicides with violent methods and nonviolent methods. Multiple logistic regression models were used to analyze related factors for violent methods of suicides. Conditional logistic regression models were used to analyze difference of data between the suicide cases and their paired controls of both the violent and the non-violent groups. For determination of statistical significance and for inclusion of variables in the model, we considered *P*<0.05 as the level of significance in all the statistical analysis process.

## Results

### (1) Characteristics of rural suicides in China

Of all the 199 cases with identified suicide methods, 84 (42.2%) were females and 115 (57.8%) were males, the male-female ratio being 1.37; 60 (30.2%) cases were diagnosed with mental disorders by SCID and 139 (69.8%) were reported to be without any mental problems.

78 (39.2%) out of all the 199 cases were classified into the violent group and 121 (60.8%) cases were classified into the non-violent group. The most common method of suicide was ingesting pesticide, with 103 (51.8%) cases dying of this method. The second method reported was hanging, with 73 (36.7%) cases. Hanging and drowning (5 cases, 2.5%) were the only two violent methods of suicide observed in this sample (showed in Table 1). 56 cases (43.2%) of the 130 impulsive suicides committed suicides with violent methods while the other 74 cases (56.9%) employed non-violent methods. Besides, in reference to the previous study by Conner and Phillips's team [22], we also categorized the suicides observed into two sub-groups: the low-planed (impulsive) suicides, with the SIS≤4; the middle and high planed (non-impulsive) suicides, with the SIS>4. The cut point was also chosen *post hoc* to obtain impulsive cases accounting for approximately one third of all cases. As a result, we obtained 62 (31.16%) impulsive suicide cases, of which 18 committed suicide with violent methods and 44 with non-violent methods. Furthermore, 39 (88.64%) of the 44 impulsive suicides with non-violent methods were killed by ingesting pesticicdes. The other 137 (68.84) cases, which accounted approximately two third out of the 199 case group, were categorized as non-impulsive suicides. What's more, majority of all the 199 suicide cases happened during daytime (142, 71.4%) and at home (165, 82.9%). Similarity was found within the cases by hanging with 58 cases (79.5%) in daytime and 66 cases (90.4%) at home, and all the 5 observed drowning cases happened outside of home. (see in Table 1)

**Table 1:** Distribution of methods leading to death of the 199 rural completed suicide cases observed in this study.

Suicide Methods	Frequency (%)	Frequency (%) by Gender	Frequency (%) by Time	Frequency (%) by Place	Frequency (%) by SCID Diagnose
		M/F	Day/Night	Home/Outside	Yes/No
Pesticides	103 (51.76)	55 (53.40)	69 (67.00)	83 (80.58)	27 (26.21)
		48 (46.60)	34 (33.00)	20 (19.42)	76 (73.79)
Other Poison	10 (5.03)	7 (70.00)	7 (70.00)	9 (90.00)	3 (30.00)
		3 (30.00)	3 (30.00)	1 (10.00)	7 (70.00)
Overdose	3 (1.51)	1 (33.33)	3 (100.00)	3 (100.00)	1 (33.33)
		2 (66.67)	0(0.00)	0 (0.00)	2 (66.67)
Suffocation	2 (1.01)	0 (0.00)	2 (100.00)	1 (50.00)	0 (0.00)
		2 (100.00)	0 (0.00)	1 (50.00)	2 (100.00)
Other	3 (1.51)	3 (100.00)	1 (33.33)	3 (100.00)	0 (0.00)
		0 (0.00)	2 (66.67)	0 (0.00)	3(100.00)
Hanging	73 (36.68)	47 (64.38)	58 (79.45)	66 (90.41)	28 (38.36)
		26 (35.62)	15 (20.55)	7 (9.59)	45 (61.64)
Drowning	5 (2.51)	2 (40.00)	2 (40.00)	0 (0.00)	1 (20.00)
		3 (60.00)	3 (60.00)	5 (100.00)	4 (80.00)
Total	199 (100)	115 (57.79)	142 (71.36)	165 (82.91)	60 (30.15)
		84 (42.21)	57 (28.64)	34 (17.09)	139 (69.85)

### (2) Factors for violent methods of suicides by univariate and multivariate logistic regression model analyses

Results of single-variable analyses are showed in Table 2. Compared to the non-violent group of which 80 cases reported, fewer cases (22 cases) of the violent group reported pesticides storage at home (28.2% vs. 66.1%, *P*<0.001). Analysis of the suicide intent indicated that the violent group had relatively higher score than the non-violent group (6.5±2.4 vs. 5.6±2.5, *P* = 0.013). In terms of mental disorders, the violent group seemed more likely to be positively diagnosed by SCID, however, no significant difference was found (37.2% vs. 25.6%, *P* = 0.083). Similar result was found in the analysis of self-esteem between the two groups, with the violent group's mean score higher than that of the non-violent group (24.2±5.8 vs. 22.7±5.3, *P* = 0.067). No other significant differences were found in the single-variable analysis process in this study (see in Table 2).

**Table 2:** Single-variable analysis of characteristics of the suicide cases between the violent group (n = 78) and the non-violent group (n = 121).

Variables	Violent Group [n (%)/mean (s.d.)]	Non-violent Group [n (%)/mean (s.d.)]	*P*
Age	61.62±19.58	60.70±16.46	0.726
Male	49 (62.82)	66 (54.55)	0.249
Education, years	3.18±3.59	2.80±3.41	0.451
Married	45 (57.69)	72 (59.50)	0.800
Personal income, RMB (Median (25^th^ and 75 Percentile))	1500.00 (0.00, 4648.40)	1000.00 (0.00, 4000.00)	0.116
Belief in Religion-yes	10 (12.82)	13 (10.74)	0.655
Season—summer or autumn	38 (48.72)	63 (52.07)	0.645
Timing—daytime	60 (76.92)	82 (67.77)	0.179
Place—home	66 (84.62)	99 (81.82)	0.609
Impulsive suicides	18 (23.08)	44 (36.37)	0.060
Negative Life Event-1 and more	71 (91.03)	104 (85.95)	0.283
Social Support (Median (25th and 75 Percentile))	35.50 (30.00, 41.00)	36.00 (30.50, 40.00)	0.758
Physical illness	46 (58.97)	79 (65.29)	0.368
Mental disorders	20 (25.64)	19 (15.70)	0.085
Pesticides storage in home	22 (28.20)	80 (66.11)	<0.001
Suicide in family	8 (10.26)	18 (14.88)	0.345
Previous attempts–1 and more	17 (21.79)	22 (18.18)	0.531
Suicide intent	6.47±2.45	5.57±2.52	0.013
Hopelessness	64.45±16.04	61.09±18.31	0.087
Anxiety	46.08±9.62	44.83±10.82	0.407
Impulsivity	45.10±17.48	42.70±18.64	0.366
Self-esteem	24.17±5.81	22.70±5.26	0.067

We used the multiple logistic regression models for analysis of factors related to violent methods of suicide in this study. After adjusting age and gender, results showed that pesticides storage at home was negatively related to violent methods. On the other hand, suicide intent was positively related to the choice of violent methods, which means that suicides with violent methods showed relatively stronger or more resolute suicide intent than suicides with non-violent methods (see in Table 3).

**Table 3:** Factors related to violent methods in rural Shandong, China: a logistic regression model (n = 199).

Variables in the Equation					
Variables	*β*	*P*	OR	95.0% C.I. for OR	
				Lower	Upper
Pesticides Storage at Home	−1.624	<0.0001	0.197	0.104	0.372
SIS	0.153	0.017	1.166	1.027	1.323
Constant	−0.606	0.168	0.546		

### (3) Factors for suicide with violent methods and without by conditional logistic regression model analyses

Furthermore, conditional logistic regression models were carried out respectively in the data of the violent group and the non-violent group together with their paired controls. Same risk factors were identified based on results of the two groups (see in Table 4–5). Interestingly, hopelessness score and negative life events were included within the final regression models for both the violent group and the non-violent group. Besides, Cochran-Armitage trend test (Z = 6.2627 and *P*<0.0001 for the violent group; Z = 8.7290 and *P*<0.0001 for the non-violent group) showed that there indeed was a positive propensity that more negative life events within 1 year before the suicide behavior would cause more risk for committing suicide.

**Table 4:** Single-variable analyses of risk factors of suicide for the violent group and the non-violent group with their paired controls.

Variables	Violent Group			Non-violent Group		
	Cases [n (%)/mean (s.d.)]	Controls [n (%)/mean (s.d.)]	*P*	Cases[n (%)/mean (s.d.)]	Controls[n (%)/mean (s.d.)]	*P*
Education years	3.18±3.59	3.90±3.98	0.239	2.80±3.41	3.39±4.10	0.221
Married	45 (57.69)	62 (79.48)	0.003	72 (59.50)	101 (83.47)	<0.001
Personal income, RMB [Median (25th and 75 Percentile)]	1500.00 (0.00, 4531.20)	2000.00 (0.00, 6000.00)	0.094	1000.00 (0.00, 4000.00)	2500.00 (0.00, 5375.00)	0.621
Belief in Religion	10 (12.82)	1 (1.28)	0.005	13 (10.74)	3 (2.48)	0.001
Negative Life Events-1 and more	71 (91.03)	38 (48.72)	<0.0001	105 (86.78)	41 (33.88)	<0.001
Social support [Median (25th and 75 Percentile)]	36.00 (30.50, 41.00)	40.00 (36.00, 45.00)	<0.0001	36.00 (30.25, 41.75)	42.00 (37.00, 45.00)	<0.001
Physical illness	46 (58.97)	33 (42.31)	0.037	79 (65.29)	48 (39.67)	<0.001
Mental disorders	20 (25.64)	1 (1.28)	0.004	19 (15.70)	1 (0.83)	<0.001
Pesticides storage at home	22 (28.21)	32 (41.03)	0.092	80 (66.12)	51 (42.15)	0.002
Suicide in family	8 (10.26)	2 (2.56)	0.049	18 (14.88)	1 (0.83)	<0.001
Hopelessness	64.45±16.04	38.72±12.98	<0.0001	61.43±17.10	38.91±10.76	<0.001
Anxiety	46.08±9.62	33.60±7.96	<0.0001	45.03±10.63	32.40±7.48	<0.001
Impulsivity	45.10±17.48	30.49±12.61	<0.0001	42.70±18.64	29.90±14.54	<0.001
Self-esteem	24.17±5.81	19.36±4.75	<0.0001	22.70±5.26	18.55±4.22	<0.001

**Table 5:** Paired case-control analyses by conditional logistic regression models for the violent group (n = 78) and the non-violent group (n = 121).

Variables in the Equation						
	Variables	*β*	*P*	OR	95.0% CI for OR	
					Lower	Upper
Violent Group	Negative Life Events	0.549	0.007	4.443	1.514	13.034
	Hopelessness	0.048	0.002	1.161	1.057	1.276
Non-violent Group	Negative Life Events	0.795	0.002	12.274	2.584	58.291
	Hopelessness	0.061	0.001	1.235	1.095	1.392

## Discussion

In this study, with analyses of valid data of the 199 suicides and their paired controls, major findings included: (1) in rural China, completed suicides with non-violent methods outweighed those with violent methods in amount; (2) the leading method of committing suicide in rural China was ingesting pesticides, and the violent suicide methods included hanging and drowning; (3) suicides choosing violent methods tended to be characterized as no pesticide storage at home and higher suicide intent degree; (4) pesticide storage at home and low suicide intent degree might be related with non-violent suicide methods; (5) negative life events, anxiety, hopelessness and former suicidal behavior of family members were found to be risk factors for deaths by violent suicide methods, while negative life events and hopelessness were risk factors leading to deaths by non-violent suicides.

Still being a forbidden topic among residents, suicide behavior in China would never end as cases kill themselves but would last long enough as a humiliating stigma for the family and even the community, which is a major cause for the underestimation of actual suicide rates and the practical reason for the informants of 19 out of all the 219 cases who were firstly recruited in our study but finally refused to take the interview.

Based on our data, suicide causes more death in male than in female, this finding is similar to previous suicide researches but different with suicides in other regions of the world. To be mentioned, though, such difference in gender ratio of suicide rates may also be caused by failing to interview those uncovered suicide cases in rural China [2], [17].

Other commonly known unique characteristics of Chinese suicides were also proved in our study. It has been noted that most Chinese suicides could not be diagnosed with any particular mental disorders [2], which is quite different from researches on populations of the west world where severe mental diseases are one of the leading causes for suicide behavior [1], [9], [48], [49]. Low planed (impulsive) suicides appeared to be quite common, with 62 cases (31.16%) of all the 199 cases categorized as impulsive suicides according to our database. The previous research on low planed suicides in rural China [22] claimed that low planed suicides tended more likely to choose non-violent methods, especially pesticide ingestion. Although no statistical correlation was found between impulsive suicides and choice of violent or non-violent methods according to our results, we could not prove the former research to be with invalid conclusions with our data showing that majority of the 62 (44 cases, 88.64%) low planed cases committed suicide by ingesting pesticide. Studies on Chinese rural suicides [2], [12], [17], [47] showed that suicide behaviors were chosen by victims under a conflicting situation with impulsivity in the purpose to intimidate or threaten someone other than to practically kill themselves. What's more, ingesting pesticide was the leading method among all cases observed. As the only two violent suicide methods observed in this study, hanging and drowning ranked the second and the forth respectively for their frequencies, and the two kinds of methods were both readily available for rural residents. Pattern of suicide methods showed in our study cohered with former similar studies [12], [16], [50]. All suicide methods observed in our study were highly used methods by suicides ever reported in rural China and no novel methods were identified.

Although non-violent methods were more common in suicides of rural China, suicides with violent methods accounted for 39.2% of all the 199 completed suicides interviewed in our study, indicating that prevention strategies targeted at violent suicide methods can never be ignored concerning the high lethality.

Access, availability, social acceptability of certain suicide method and some location-specific factors such as access to firearms or tall buildings are important factors responsible for the prevalence of that method. Our findings supported such theory and found practical evidence. As a country with a large proportionate of rural population, China witnesses approximately 1.4 million tons of pesticides each year [12], [17], [47], and this is closely related to the fact that ingesting pesticide is the most favored method for suicide in rural China.

As the only two violent methods observed in this study, hanging and drowning are not only readily feasible in rural China but also adherent with the traditional Chinese culture of holding a wholesome body even after death. Semi-structured interviews showed that most hanging suicides in our study happened at home on the major beam inside the house or on the big branches of trees in the home yards. Under such circumstances, suicide victims could easily complete the suicide without immediate disturbance even during daytime. Meanwhile, most suicides by drowning happened in the rivers running through the countryside or in the dead waters formulated by over-heavy rains, which are both quite common environments in rural China. Analyses of our database showed that pesticide storage at home was negatively correlated with violent suicides, which is another evidence supporting the accessibility and availability of certain suicide methods would present much propensity to such methods while facing different choices. On the other hand, though, further confirmation is needed to clarify the influence of suicide intent and pesticide storage at home on the choice of non-violent methods, with the hypothesis that these two factors might show positive effect on non-violent methods in a future research with a larger sample. Conformation of such hypothesis would offer practical support in suicide prevention programs such as urging for safe storage and usage of pesticides in rural China.

What's more, ingesting poison, hanging, and drowning were also widely known by most people as suicide methods with a long history in rural China, with a lot of cases either reported by the media or generally told by local residents. All these affirmed that awareness of certain methods and the so-called copy-cat or imitation effects play as important determinants for how to commit a suicide, which had also been supported by other researches claiming that media reports of new suicide methods would cause increase of suicide cases by such new methods [7], [21]. Although there is still no instruments to precisely evaluate the influence of factors such as availability and social-cultural acceptability of certain methods, former studies [7], [51], [52] found evidence supporting such hypothesis as publicity of suicide methods and traditional view of deaths would affect the choice of committing suicide.

There were not quite many kinds of violent suicide methods observed in this study other than hanging and drowning, though another study [12] focused on violent suicide of young rural Chinese found some cases of wrist-cutting, electric shock, and jumping from high places.

Some reported studies [10], [15] on suicide behavior found that increasing of age was negatively related to suicides with violent methods, yet our results in this study did not found significant relationship between the age and methods, with the similar age distribution between the violent group and the non-violent group. Former European studies [6], [9] found statistical difference between men and women that men tended more likely to undertake more lethal methods while committing suicide, which was also not supported by our data.

Furthermore, despite the fact that substantial use of pesticides in rural area has pronounced seasonality according to the agriculture work, we did not find statistical relationship between suicides by violent methods and seasonality of suicide behavior, which also differs from findings of other researches [12], [16]. Other suicide behavior features including timing (Day/Night), or place (Home/Outside) were also not identified as factors of significance for violent suicide methods in our study, which were quite different from other researches [12], [16].

Former studies [6], [9], [11] on suicide methods found that psychological status before committing suicide would affect victims' choice of different methods. Different from Jia & Zhang's research [12] of young suicides in rural China, according to our data, higher suicide intent score, widely viewed as a convincing marker of the resolution to death of a suicide case, was positively related to the choice of violent methods while committing a suicide. However, our study found no other statistically significant psychological factors apart from suicide intent that may influence the choice of suicide methods while comparing data between the violent group and the non-violent group cases.

Belief in religion was proved to be a protective factor for suicide in western communities [1], [6], [13], [48] and a risk factor for suicide in rural China [2], [3], [17], but our results found no influence of religion on neither the choice of certain suicide methods nor the idea of committing suicide. Our data found a relatively low incidence of mental illnesses among suicides, similar to former studies among Chinese populations [2], [3], [17], [53]. Although identifying and preventing mental and psychological problems have long been established as practical strategies for suicide prevention [11], [13], [48], [54], diagnose results of mental disorders or other psychological problems by SCID and other psychological scales were not included in the final equation according to the multiple logistic regression analysis between suicides with the violent methods and without. Nevertheless, separate conditional logistic regression analysis for data of the cases and their paired controls of the violent and the non-violent group did show different results with respect to mental and psychological risk factors of suicide, indicating such relationships remained unclear according to our data and more researches were needed for confirmation.

The ultimate goal for suicide study would be feasible and effective prevention or intervention strategies. Various kinds of measurements to avoid accessibility of pesticides or other poisons have been implemented or suggested in some Asian areas and outcomes have been evaluated [21], [55], [56]. However, it is also important to be aware that while talking about intervention strategies specifically designed for different methods, none of other observed or possible methods should ever be left without intervention. It has been discussed that with restriction of a certain suicide method being reinforced, a replacement effect [7], [12], [21], [48], [52], [56] may show up as suicides by other methods correspondingly increase, though a study in Denmark [13] claimed that restriction of lethal suicide means such as firearms resulted in apparent decline in the overall suicide rates by all means as well as suicides by gunshots.

According to the conditional logistic regression analyses of risk factors for completed suicide respectively among the violent group and the non-violent group with their paired controls, negative life events and high hopelessness scores were included in the final regression models for both groups as risk factors for suicide deaths. Therefore, with the speculation that negative life events and extent of hopelessness are risk factors for completed suicides regardless of certain methods, much work should be aimed in improving the living conditions of rural residents who are experiencing miseries or overwhelming life changes. Besides, local authorities should welcome external charity funding organizations to offer financial and medical help to residents who are suffering.

Our study had both strengths and limitations. Firstly, sample of this research is relatively limited and some results may not be qualified to extrapolate to the whole population. Suicide cases whose informants refused to participate the interview or possible suicide cases who had been unreported or reported as deaths by other reasons than suicides may, and the limitation of research counties recruited may affect results such as the gender ratio and other data manifestation. Secondly, since this was a paired case-control study, many more researches, especially prospective longitudinal programs need to be done in the future for confirmation of certain unclear areas. Also, methodological alternative of control selection such as involving deaths by other causes may show different effects from recruiting controls who were still alive by the time of interview. Thirdly, concerning that psychological autopsy is a retrospective method, and our interview subjects were informants for completed suicide cases, information bias including recall mistakes or uncertain emotional propensity of informants toward the suicide victims may exist, though we have tried our best to keep the biases within controllable limits. Prevention strategies were discussed but future implementation and evaluation are needed to test the feasibility and outcomes.

However, our study does have substantial values and advantages. This was a pioneering research program conducted in rural China focused specifically on violent and non-violent methods among all age groups and both genders. Research methods and results showed special significance for reference. Comprehensive information including physical and mental status of the suicides were obtained and analyzed. Both consistency and inconsistency compared with previous suicide studies were mentioned, which are all of much value for future researches. Possible prevention and intervention suggestions were discussed, some of which had been proved to be substantially effective in other countries and all were meticulously brought up by our research team.

## Supporting Information

File S1 **Database for violent & non-violent suicides.**
(XLS)
